# SAXS Studies of TiO_2_ Nanoparticles in Polymer Electrolytes and in Nanostructured Films

**DOI:** 10.3390/ma3114979

**Published:** 2010-11-22

**Authors:** Aleksandra Turković, Pavo Dubček, Krunoslav Juraić, Antun Drašner, Sigrid Bernstorff

**Affiliations:** 1 Institute “Ruđer Bošković”, P.O. Box 180, HR-10002 Zagreb, Croatia; E-Mails: dubcek@irb.hr (P.D.); kjuraic@irb.hr (K.J.); drasner@irb.hr (A.D.); 2 Sincrotrone Trieste, ss. 14, km 163, 5 Basovizza, 34012 Trieste, Italy; E-Mail: bernstorff@elettra.trieste.it (S.B.)

**Keywords:** polymer electrolytes, solar cells, nanoparticles TiO_2_, nanostuctured films, GISAXS, SAXS/DSC/WAXD

## Abstract

Polymer electrolytes as nanostructured materials are very attractive components for batteries and opto-electronic devices. (PEO)_8_ZnCl_2_ polymer electrolytes were prepared from PEO and ZnCl_2_. The nanocomposites (PEO)_8_ZnCl_2_/TiO_2_ themselves contained TiO_2_ nanograins. In this work, the influence of the TiO_2_ nanograins on the morphology and ionic conductivity of the nanocomposite was systematically studied by transmission small-angle X-ray scattering (SAXS) simultaneously recorded with wide-angle X-ray diffraction (WAXD) and differential scanning calorimetry (DSC) at the synchrotron ELETTRA. Films containing nanosized grains of titanium dioxide (TiO_2_) are widely used in the research of optical and photovoltaic devices. The TiO_2_ films, prepared by chemical vapor deposition and e-beam epitaxy, were annealed in hydrogen atmospheres in the temperature range between 20 °C and 900 °C in order to study anatase-rutile phase transition at 740 °C. Also, grazing-incidence small angle X-ray scattering (GISAXS) spectra for each TiO_2_ film were measured in reflection geometry at different grazing incident angles. Environmentally friendly galvanic cells, as well as solar cells of the second generation, are to be constructed with TiO_2_ film as working electrode, and nanocomposite polymer as electrolyte.

## 1. Introduction

In order to study nanocomposite polymer electrolytes and nanophased metal-oxide films on glass substrate, suitable techniques have been applied to obtain accurate measurements of the structure on an atomic, as well as on a medium, range scale. Small-angle X-ray scattering (SAXS) experiments generally fulfill these requirements; irradiate a sample with X-rays, measure the resulting scattering pattern, then determine the structure that caused the observed pattern. Scattering patterns are caused by the interference of secondary X-ray waves that are emitted from electrons, when irradiated. Scattering of X-rays is caused by differences in electron density. Since the larger the diffraction angle, the smaller the length scale probed, wide angle X-ray diffraction (WAXD) is used to determine crystal structure on the atomic length scale while SAXS is used to explore structure on the nanometer scale.

SAXS experiments are suitable to determine the microstructure of nanocomposite polymer electrolyte. Solid electrolyte poly(ethylene oxide) (PEO) is one of the most extensively studied systems due to its relatively low melting point and glass transition temperature Tg; its ability to play host to varied metal salt systems at a range of concentrations; and to act as a binder for other phases. For these reasons PEO has been the basis of many investigations in the area based on composites of a polymer and an insulating ceramic. Polymeric complexes of (PEO)n with ZnCl_2_ have been used, due to their stability and very high conductivity as compared to other complexes [1,2]. The mechanical properties of amorphous PEO-based electrolytes are poor and attempts to improve these have included the addition of inert filler. We intended to improve the electrical conductivity of the polymer electrolyte (PEO)_8_ZnCl_2_ by introducing TiO_2_ nanoparticles [3]. Since a polymer is a composite of an amorphous and a crystalline part, and conductivity occurs in the amorphous part, this treatment was directed towards the inhibition of the crystalline phase in the polymer matrix.

Films containing nanosized grains of titanium dioxide (TiO_2_) are widely used in the research of mainly optical, photovoltaic, photo chromic and electro chromic devices. Besides, due to the size and structure of the grains, their specific applications are also determined by their porosity. In most cases there is a desirable degree of porosity which leaves the outer and inner surface of the film large enough. These morphological characteristics of TiO_2_ films depend on the method of preparation, but also on the subsequent processing of the material. Specifically, during the thermal annealing at higher temperatures, the changes in porosity and grain size, but also in the grain structure, take place, because TiO_2_ exists in three different phases (anatase, rutile and brookite) which are stable at different temperatures. Furthermore, the atmosphere during annealing can influence the stoichiometry of TiO_2_.

The aim of the present investigation was to study the structural and calorimetric behavior of (PEO)_8_ZnCl_2 _electrolyte, which was prepared as a nanocomposite using 10% of nanosized Degussa P-25 TiO_2_. The morphology of the nanocomposite films was also studied by optical microscopy. The ionic conductivity was measured with a custom-made impedance meter. The results of both methods are presented and compared with those obtained by simultaneous SAXS/DSC/WAXD measurements in order to explain the nanostructure behavior during the phase transition of polymer electrolyte to the super ionic phase above ~65 °C. The introduction of TiO_2_ nanograins and the subsequent irradiation with γ-rays of 309 KGy was performed with the intention to decrease the phase transition temperature and to increase the conductivity of the polymer electrolyte, in order to obtain properties which would be preferable for using this nanocomposite as electrolyte in the construction of galvanic or solar-cells [4,5,6,7]. 

In order to determine evolution of the grain size and specific surface area of TiO_2_ thin films on glass substrates during the phase transition from anatase to rutile phase at 740 °C, we have performed GISAXS measurements. Two different preparations of the TiO_2_ films—obtained by chemical vapor deposition (CVD) and e-beam epitaxy—were studied in order to obtain the best parameters for the grain sizes and porosity for the construction of efficient dye-sensitized solar cells based on TiO_2_ working electrodes [6,7].

## 2. Experimental Section

The polymer-salt complex was prepared by dissolving ZnCl_2_ (p.a. Merck) and poly(ethylene oxide) (Laboratory reagent, BDH Chemicals Ltd., Poole, England, Polyox WSR-301, MW = 4 × 10^6^. Prod 29740), in 50% ethanol-water solution in stoichiometric proportions. The preparation was performed by stirring nanometer sized grains of TiO_2_ (Degussa P25) into solution, so that the content of TiO_2_ was 10 weight percentage. The polymer-salt complex solution was then poured onto a Teflon plate and allowed to dry in air. The film was evacuated to 10^−6^ mbar for a few days to allow traces of the solvent to evaporate. In order to protect the film from moisture in the air during longer periods, it was stored in desiccators filled with silica gel. 

Simultaneous SAXS, WAXD and DSC measurements were performed at the Austrian SAXS beamline at the synchrotron ELETTRA, Trieste [8]. The photon energy of 8 keV was used, and the size of the incident photon beam on the sample was 0.1 × 5 mm (h × w). For each sample, SAXS and WAXD patterns were measured simultaneously in transmission setup using two 1D single photon counting gas detectors. Sample-to-SAXS detector distance was 1.75 m, corresponding to a q-range of 0.007–0.32 Å^−1^. The WAXD detector covered a d-spacing range of 0.32–0.94 nm.

The scattering wave vector s equals s = 2sinθ/λ = q/2π, where 2θ is the scattering angle and λ = 0.154 nm the used wavelength. The method of interpreting the SAXS scattering data is based on the analysis of the scattering curve, which shows the dependence of the scattering intensity, I, on the scattering wave vectors. 

The in-line micro-calorimeter built by Ollivon *et al*. [9] was used to measure simultaneously SAXS/WAXD and high sensitivity DSC from the same sample. The DSC phase transition temperature was determined at the intersection of the tangent to the peak and the baseline. The heating and cooling cycles were performed at controlled rates of ½ °C/min. Thus, the recording of one heating‑cooling cycle took 320 min to cover the ramp (20 °C→100 °C→20 °C).

Thin films of TiO_2_ have been prepared by two different methods. One set of TiO_2_ samples was prepared by e-beam epitaxy of titanium dioxide onto glass substrates. The deposition was done in a Varian 3117 evaporator under pressure of 1.33 × 10^−5^ mbar. The second way of obtaining TiO_2_ films was by the CVD method from commercial (Merck) TiCl_4_. It was deposited on the glass and quartz support at 200 °C in a homemade apparatus.

Grazing-incidence small-angle X-ray scattering (GISAXS) spectra for each TiO_2_ film were measured in reflection geometry at eight different grazing incidence angles. In this case, the path of the X-ray through the film is much longer than for standard transmission geometry. GISAXS intensity curves were obtained from the pattern recorded by a two-dimensional charge-coupled device (CCD) detector from Photonic Science (with image sizes of 1024 × 1024 pixels). The samples were mounted on a stepper motor controlled tilting stage with a step resolution of 0.001°. The camera length of the set-up was 2 m. For the angular (s-scale) calibration of the camera, rat-tail tendon was used. The data were stored in 12 bit-TIFF format. Afterwards the GISAXS images were analyzed using the IGOR software from WaveMetrics.

The morphology of the polyelectrolyte and nanocomposite films was studied using a Leitz Orthoplan optical microscope. The magnification was 20 x; polarized light was used.

Impedance measurements were performed with an impedance meter, built in our laboratory, in the frequency range from 0.1 Hz to 3 MHz. The impedance spectra were collected at a potential of 300 mV.

## 3. Results and Discussion 

### 3.1. SAXS on Nanostructured Materials 

Nanostructured materials such as nanophased films and nanocomposites such as (PEO)_8_ZnCl_2_/TiO_2_, can be considered as aggregates containing nanoparticles or nanograins [10,11,12,13,14,15]. In this case, the SAXS is caused by the difference of electron density within and around the nanoparticles. Using the Guinier approximation [16]—the scattering in the very small angle range is of Gaussian form, independent of the shape of the present particles—the sizes can be readily determined. The Porod approximation [17] is suitable to determine the specific surface area of nanostructured thin films. At high intensity synchrotron light sources, the scattered intensity is high enough that we can apply both approximations and obtain all relevant parameters. 

In this section, the outline of calculations in Guinier approximation is given for (PEO)_8_ZnCl_2_. Previously it was successfully applied on a number of metal oxides such as TiO_2_, CeO_2_, V_2_O_5_, and Ce/Sn, V/Ce mixed oxides films [6,7,10,11,12,14,15].

**Figure 1:**
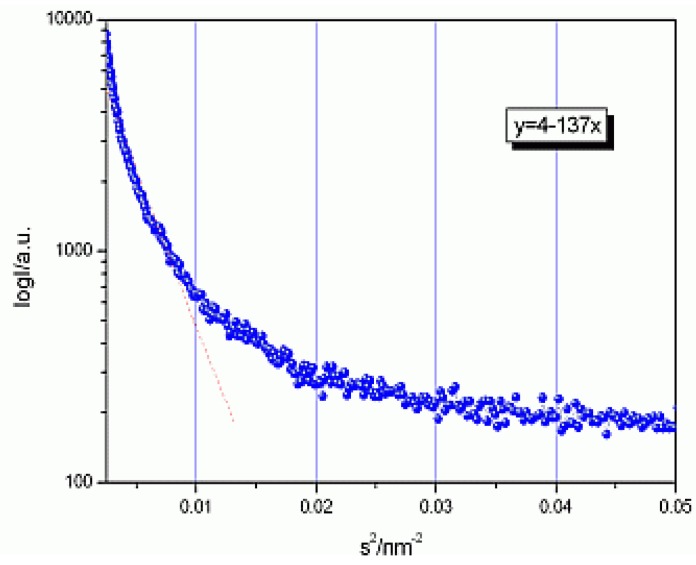
Linear fit: y = 4–137x to log (I) = f(s) for SAXS data for (PEO)_8_ZnCl_2_ at room temperature.

Figure 1 represents the data for (PEO)_8_ZnCl_2_ at room temperature (25 °C), in a *log(I) vs. f(s^2^),* *s = 2θ/λ,* plot as a test as to whether one can apply the above mentioned Guinier law:

(1) $$I(s)={\left(\Delta \rho \right)}^{2}{exp}^{\left(-R_{g}^{2}s^{2/3}\right)}$$

for small s. The "average particle radii" can be estimated from the radius of gyration Rg in the Guinier formula. They were calculated from the slopes in the linear fit of *log(I) vs. f(s^2^),* (rad). From these fitting lines we have obtained Rg and average particle radius R using R= (5/3)^1/2^ Rg (for spherical shape).

For WAXD the diameter of the nanocrystalline grains is obtained by the Debye-Scherer equation:

(2) $$D=\frac{0.9•\lambda }{\beta •\cos(\theta )}$$

where λ is the wavelength of the incident X-ray beam, and β is the full width at half maximum (FWHM) of the WAXD line.

### 3.2. SAXS/DSC/WAXD of Polymer Electrolytes, Nanocomposites of (PEO)_8_ZnCl_2_

**Figure 2:**
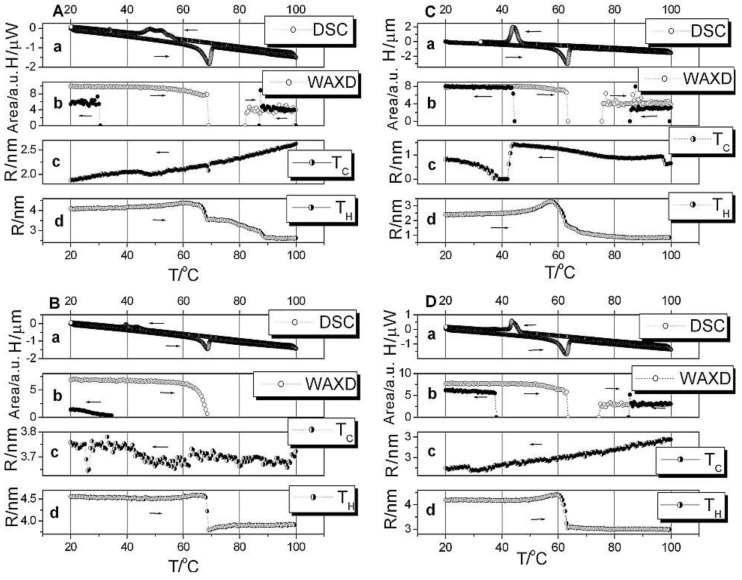
SAXS, DSC and WAXD results for samples A, B, C and D.

Figure 2 shows the results from the simultaneous SAXS, DSC and WAXD measurements in the temperature range from 20 °C to 100 °C to 20 °C at rate of ½ °C/min on the polymer electrolytes: (PEO)_8_ZnCl_2_, (PEO)_8_ZnCl_2_/TiO_2_, (PEO)_8_ZnCl_2_ irradiated with a dose of 309 KGy and (PEO)_8_ZnCl_2_/TiO_2_ + 309 KGy (denoted as A, B, C and D, respectively). The evolution of the average radii of grain sizes obtained by applying equation (1) is compared to the corresponding DSC and WAXD spectra behavior. The hysteresis is present in the heating-cooling cycle. 

In Figure 2 graph A shows the results from the measurements in the temperature range from 20 °C to 100 °C to 20 °C at a rate of ½ °C/min on polymer electrolyte (PEO)_8_ZnCl_2_. The intensity close to Is (for s = 0) falls at 68.3 °C indicating the phase transition temperature in the heating cycle. The phase transition temperature in the cooling cycle is at 47.6 °C due to hysteresis. The average radius of grains varies from 4.0 nm to 4.4 nm in the region below the phase transition temperature and then from 3.5 nm to 2.6 nm in the highly conductive phase. The cooling cycle in the SAXS data shows a change of grain sizes in the range from 2.6 nm to 1.9 nm. SAXS measurements for (PEO)_8_ZnCl_2_/TiO_2_ (Figure 2B), result in changes of grain sizes from 4.6 to 3.7 nm; the third sample (PEO)_8_ZnCl_2_ irradiated with a dose of 309 KGy (Figure 2C), registers changes from 3.3 to 0.7 nm and during the fourth run for the sample (PEO)_8_ZnCl_2_/ TiO_2_ + 309 KGy (Figure 2D), grain sizes change from 4.4 to 2.7 nm. From these we can generally conclude that the average grain sizes in all four samples remained in the same range from 0.7 to 4.6 nm.

In a lamellar picture of PEO [18], these grain sizes would correspond to the lamellae LP2 with no integrally folded (NIF) chains [19] combined with salt and TiO_2_. 

The SAXS, WAXD and DSC data show a hysteresis, *i.e*., phase transition temperature in the cooling cycle is much lower than 65 °C. This temperature is the melting temperature of the PEO crystallites, *i.e.*, spherulites [20]. In the case of the nanocomposite polymer electrolyte, combined forms of PEO and ZnCl_2_, or both, in combination with TiO_2_ crystallites, influence the melting temperature. The combined WAXD, SAXS and DCS results are summarized in Table 1.

**Figure 3:**
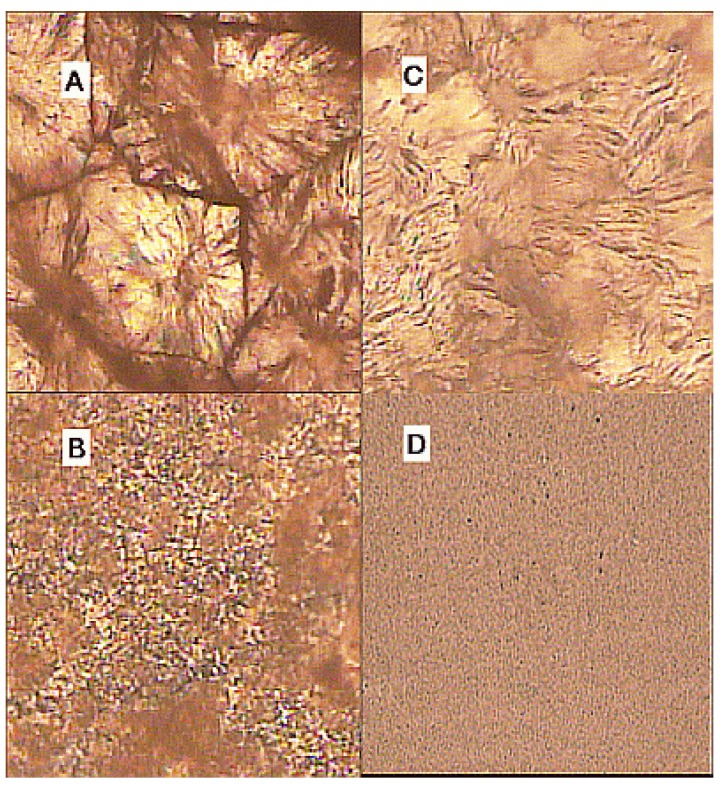
Optical microscopy pictures for samples A, B, C and D with a magnification of 20 x at room temperature [20].

Figure 3 shows optical microscope pictures for samples A, B, C and D, taken by a Leitz orthoplan optical microscope in polarized light and with magnification of 20x. These pictures are taken at room temperature and are presented here to visually support the SAXS/DSC/WAXD data of Figure 2. In optical micrographs of unirradiated (PEO)_8_ZnCl_2_ film (Figure 3A), spherulites that are impeding Zn^2+^ ion-based conductivity, are clearly visible. Addition of TiO_2_ nanograins reduced the crystallinity, such that in nanocomposites prepared from unirradiated PEO, the spherulites are very small (Figure 3B). In the course of crosslinking polymer chains, the space for spherulite growth is reduced; thus in films prepared from irradiated PEO, these organized structures are reduced (Figure 3C). In the nanocomposite (PEO)_8_ZnC_l2_/TiO_2_ prepared from irradiated PEO, spherulites are not visible (Figure 3D). 

**Table 1:** Changes of average grain radius R (nm) calculated by (1), R = D/2 as determined from (2) and phase transition temperatures t (in °C) in (PEO)_8_ZnCl_2_/TiO_2_ nanocomposite, polyelectrolyte during heating and cooling with rate of ½ °C/min as determined by SAXS/WAXD/DSC measurements.

**Sample**	**Heating**				
	**SAXS**		**WAXD**		**DSC**
	*t (°C)*	*R (nm)*	*t (°C)*	*R (nm)*	*t (°C)*
**A**	68.3	4.0–4.4 3.5–2.6	68.9; 82.2	34–45; 95–96	65.3
**B**	68.7	4.6–4.5 3.8–3.9	68.9	35–47	65.0
**C**	62.5	2.4–3.3 1.7–0.8	63.0; 75.5	45–51; 82–82	59.0
**D**	63.4	4.2–4.4 3.0–3.0	63.4; 74.7	45–58; 109–111	58.4
**Sample**	**Cooling**				
	**SAXS**		**WAXD**		**DSC**
	*t (°C)*	*R (nm)*	*t (°C)*	*R (nm)*	*t (°C)*
**A**	47.6	2.6–2.0 2.0–1.9	30.3; 87.0	57–61; 68–85	56.2
**B**	49.2	3.7–3.65 3.65–3.7	35.0	50–102	44.6
**C**	42.2	0.7-1.5 0.8–0.7	43.0; 85.6	45–45; 109–111	46.6
**D**	28.8	2.9–2.8 2.7–2.7	37.6; 85.1	54–62; 66–111	46.4

**Legend : **A = (PEO)_8_ZnCl_2_, B = (PEO)_8_ZnCl_2_/TiO_2_, C = (PEO)_8_ZnCl_2_ + 309 KGy, D = (PEO)_8_ZnCl_2_/TiO_2_ + 309 KGy

**Figure 4:**
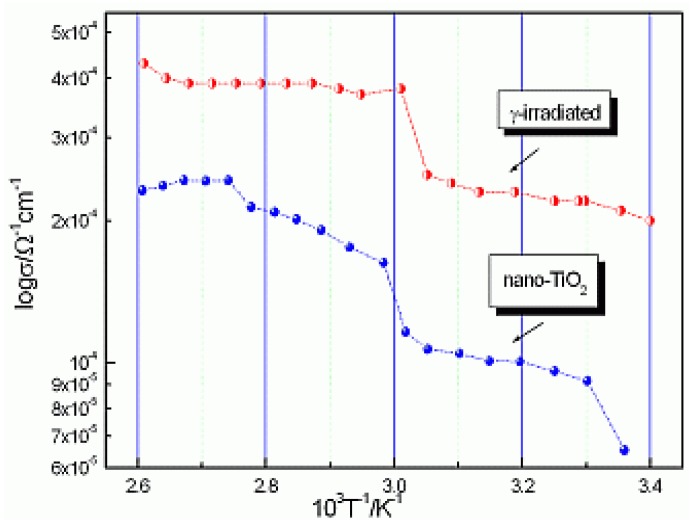
Plot of log (σ) *vs*. 1000/T for γ-irradiated and nanocomposite polymer electrolyte [3].

In our previous measurements by impedance/admittance spectroscopy, performed with Zn nonblocking electrodes [3], a steep increase of ionic conductivity σ of the polyelectrolyte film, proportional to the irradiation dose, was observed. The transition temperature to the superionic phase that occurs due to melting of spherulites decreases. The conductivity of polymer electrolyte prepared by irradiation crosslinking of PEO using 309 KGy was the largest. Nanocomposite polymer electrolytes were easy to handle and formed a compact film as opposed to the poor mechanical properties of polymer electrolyte prepared with irradiated PEO. The nanocomposite prepared from irradiated PEO exhibited an order of magnitude higher room temperature conductivity and a two- order of magnitude higher conductivity at the transition temperature than the corresponding polyelectrolyte film without TiO_2_, as shown in Figure 4.

The combination of the SAXS/DSC/WAXD methods reveals the nature of the physical transformation of the polymer electrolyte into a super ionic conductor. The nanocomposite crystalline and amorphous polymer matrix turns into an amorphous highly conductive phase. Whereas previously, using measurements with faster rates of 1 °C/min, 3 °C/min and 5 °C/min [21], WAXD exhibited lines and thus crystalline grains only in the low temperature crystalline phases, here, with the slower rate measurements of ½ °C/min, crystalline lines also exist at higher temperatures (82.2 °C–100 °C and 100 °C–87 °C, heating and cooling respectively, for sample A). Small intensity peaks at higher temperatures in WAXD, which are slightly shifted, indicate crystallinity of combined PEO/ZnCl_2_ and PEO structures in a liquid like amorphous phase [2,22].The exception of this is sample B, which is nanocomposite, and has a completely amorphous WAXD phase at high temperature. The different morphology between treatments by irradiation, and by introducing TiO_2_ nanograins, can be observed in Figure 3. As can be seen in Figure 4, the crystalline forms in the high temperature phase increase the conductivity as a difference in the conductivity between nanocomposite and irradiated polymer electrolyte. 

The influence of morphology on the conductivity of the nanocomposite could be explained by the effect of confinement on polymer mobility [23]. Dispersion of polymer nanospheres in a medium [24] or of nanoparticles in polymer matrices [25], are examples of confinement of polymer chains. The behavior of polymer fluids in a restricted space can be very different from in bulk, especially when the molecules are confined to dimensions comparable to their sizes. The equivalence in the behavior between polymer nanocomposites and thin polymer films has recently been quantitatively verified for silica/polystyrene nanocomposites [26]. In our case TiO_2_ nanograins are forming confinement for PEO chains. The higher the percentage of confined PEO, the faster is the ion mobility. This should be related to the noncrystalline structure of the confined PEO. Also, the interactions of the anion with nanograins result in increased mobility of the cation [27]. In the irradiated polymer electrolyte, PEO could crystallize at higher temperatures, as there is no TiO_2_ confinment to prevent it. 

SAXS shows the existence of nanograins in both the low and high temperature phase in all samples. At the phase transition temperature, the grain size changes; it becomes smaller at higher temperatures. The nature of the nanograins as seen by SAXS is not just the pure crystalline, but also the partly amorphous form, while WAXD records only pure crystalline nanograins. Thus the picture of the highly conductive phase consists of a completely amorphous or liquid-like polymer matrix, which is known to be suitable for ion-conduction by elastic movement of PEO chains, and of crystalline PEO/ZnCl_2_ and PEO structures [2], which could also contribute to Zn^2+^-ion conduction by a hopping mechanism. Small intensity peaks at high temperatures recorded by WAXD for nonirradiated and irradiated polymer electrolyte support the idea of a liquid-like phase with crystalline nanograins between which hopping could occur. Nanocomposites exhibit high conductivity by PEO chains confinement mechanism. Under proper circumstances, the presence of ion-transport pathways can be as important as the polymer segmental motion [28,29].

### 3.3. GISAXS of TiO_2_ Nanophased Films Obtained by CVD and E-Beam Epitaxy

TiO_2_ thin film on a glass substrate can be considered as an aggregate containing TiO_2_ nanosize grains and nanosize pores [10,11,12,13,14,15]. In this case, SAXS is caused by the difference of electron density within and around the nanosize grains. Using the Guinier approximation [16], the grain size can be readily determined. The limiting angle Θm of the small-angle diffuse scattering is approximately Θm = λ/2D, where D is the largest grain diameter. The Porod approximation [17] is suitable for determining the specific surface area of nanosized thin films. In our previous measurements [10] with a laboratory X-ray source, the intensity of the recorded signal was too small to resume the part of the scattering curve relevant for the Porod approximation. With high intensity synchrotron radiation sources, the scattered intensity is high enough so that we can apply both approximations and obtain both relevant parameters, that is, grain size and the specific surface of TiO_2_ thin films.

**Figure 5:**
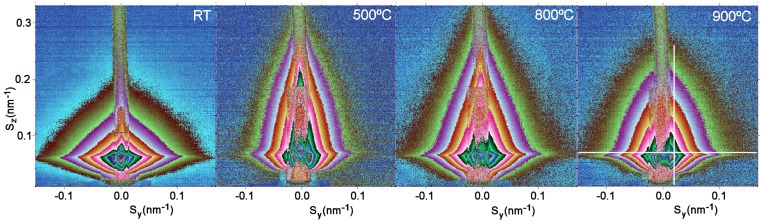
2D GISAXS patterns of CVD obtained TiO_2_ films. The white lines indicate where the 1-D cuts for the estimation of the “R” values (shown in Figure 7) were taken.

**Figure 6:**
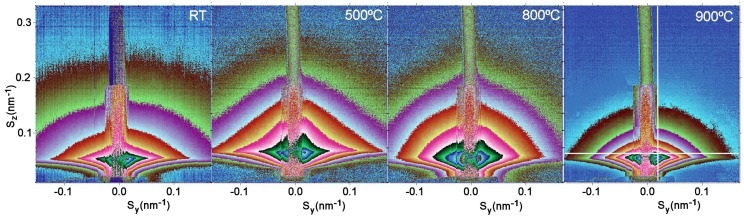
2D GISAXS patterns of e-beam obtained TiO_2_ films. The white lines indicate where the 1-D cuts for the estimation of the “R” values (shown in Figure 8) were taken.

The CVD obtained and e-beam evaporated nanophased films were annealed in hydrogen atmosphere at temperatures of 20 °C, 100 °C, 200 °C, 500 °C, 700 °C, 740 °C, 800 °C, 900 °C and 900 °C (for 5.5 hours). Figure 5 and Figure 6 show 2D GISAXS patterns of CVD films and e-beam evaporated films with four of the above mentioned temperatures. These four temperatures 20 °C, 500 C°, 800 °C and 900 °C best represent the changes in the 2-D pattern shape due to the phase transition at 740 °C. In Figure 5 CVD 2D-patterns are elongated in a horizontal direction and have a triangular shape, while after phase transition these shapes became more spherical. The shapes of e-beam evaporated films in Figure 6 are more spherical and above the phase transition, they also have spherical shapes, but the changes of intensities as a function of angle are steeper. The recorded data are reported in terms of the total scattering vector, with its horizontal (Sy) and vertical (Sz) components.

**Figure 7:**
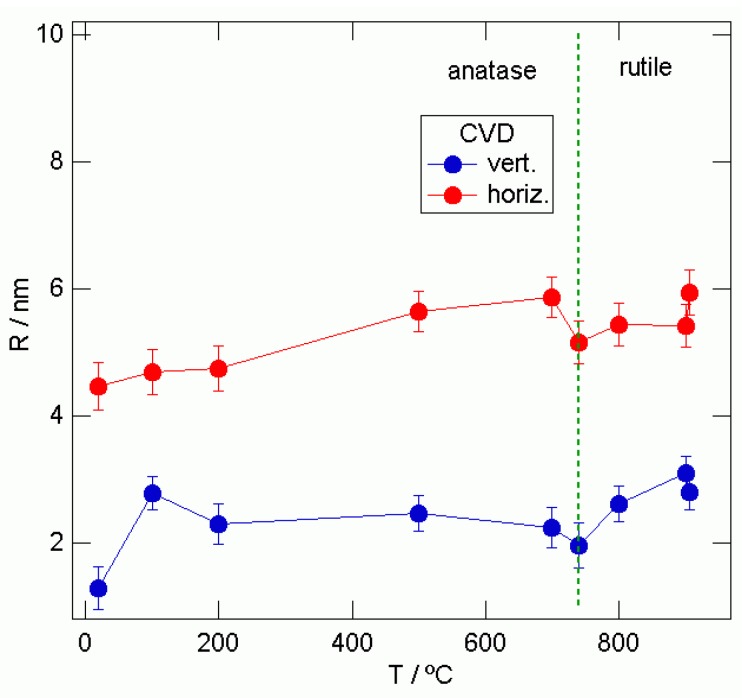
“R” values obtained fitting the vertical and horizontal 1D-cuts for the different sample annealing temperatures taken from the CVD obtained TiO_2_ films.

**Figure 8:**
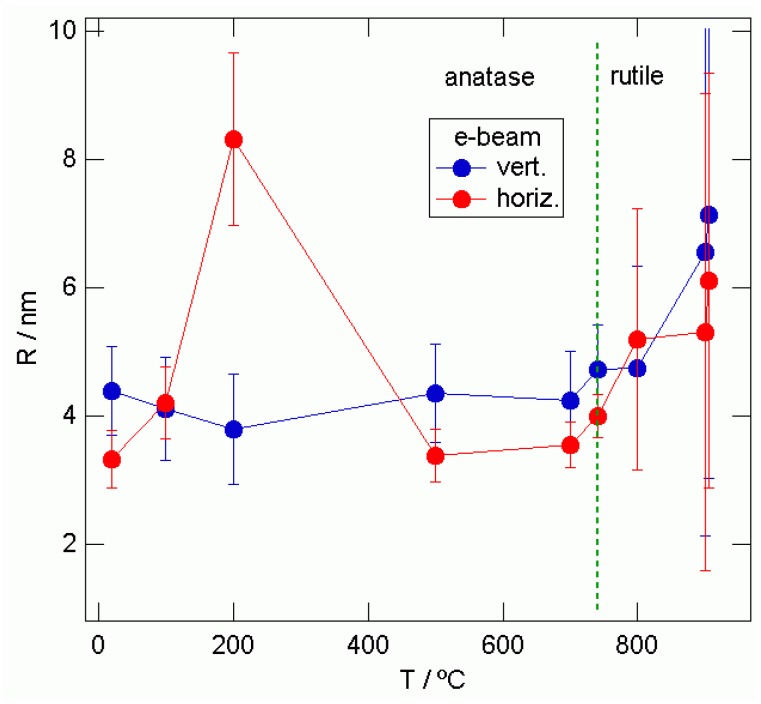
“R” values obtained fitting the vertical and horizontal 1D-cuts for the different sample annealing temperatures taken from the e-beam epitaxy obtained TiO_2_ films.

Vertical and horizontal 1D cuts were taken from the 2D-GISAXS patterns as indicated by the white lines in Figure 5 and Figure 6. Using the Guinier function yielded the average grain sizes “R” shown in Figure 7 and Figure 8. 

For the CVD samples, it is evident that the “R” values are bigger in the direction parallel to the sample surface, as in the direction perpendicular to the sample surface; Figure 7. In the anatase phase of the CVD samples, average grain sizes “R” are in the range from 4.5 nm to 5.9 nm and in the rutile, they change from 5.1 nm to 5.9 nm as obtained from the horizontal cuts. In the perpendicular direction obtained from the vertical cut, smaller values of 1.3 nm to 2.5 nm in the anatase and 1.9 nm to 2.8 nm in the rutile phase were obtained. That means that the shape of these nanograins is elongated in the direction parallel to the sample surface. The results for “R”, for the samples obtained by e-beam evaporation, are closer to spherical nanograin shapes.

From Figure 8, in the anatase phase, grain sizes “R” range from 3.3 nm to 8.3 nm and in the rutile phase from 4.0 nm to 6.1 nm in the horizontal direction. In the vertical direction, grain sizes “R” range from 4.4 nm to 4.3 nm, and in the rutile phase from 4.3 nm to 7.2 nm. For both curves, we can see two trends: first an increase of the grain size until 740 °C. Second, after the anatase‑rutile phase transition, again the grain size increases with annealing temperature.

The “R” values in the horizontal direction are closer to those for the vertical direction for the TiO_2_ films prepared by e-beam evaporation than are the CVD obtained samples. That means that the shape of these nanograins is almost spherical. For both curves, we can again see two trends, first an increase of the grain size until 740 °C and a second increase after the anatase-rutile phase transition took place. The changes of average grain radius “R” for CVD and e-beam epitaxy obtained TiO_2_ films on the glass substrate, determined by 2D-GISAXS measurements, are presented in Table 2. 

The specific applications of nanophased TiO_2_ films are also determined by their porosity. A desirable degree of porosity leaves the outer and the inner surface of the film large enough for contact of the dyes in the case of dye sensitized solar cells.

**Figure 9:**
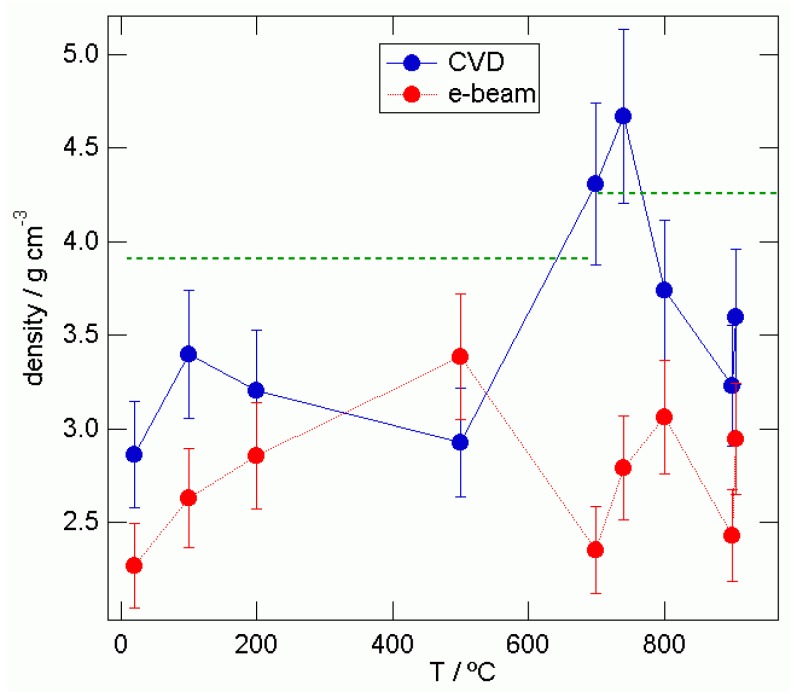
The densities of CVD and e-beam epitaxy obtained samples in anatase and rutile phase.

Here we present the differences of densities from our two preparation methods to the normal density of anatase and rutile, leaving space for the porosity of the material. This is shown in Figure 9, when comparing the densities obtained by analysis of the critical angles in the SAXS data to the bulk densities of the TiO_2_ samples (ρanatase = 3.89 gcm^−3^and ρrutile = 4.25 gcm^−3^).

The CVD prepared samples express different behavior than the samples obtained by the e‑beam epitaxy. The dotted line represents bulk densities of the anatase and rutile phases of TiO_2_ films.

**Table 2:** Changes of average grain radius R/nm calculated by (1) and densities ρ/gcm^−3^ obtained from critical angle in 1-D GISAXS obtained by horizontal cut for CVD and e-beam epitaxy obtained TiO_2_ films on the glass substrate determined by 2D-GISAXS measurements.

**Sample TiO_2_**	**GISAXS**									
	**T*(°C)***	20	100	200	500	700	740	800	900	900
**CVD**	Horizontal R/nm	4.8 ± 0.4	4.7 ± 0.4	4.7 ± 0.4	5.7 ± 0.3	5.9 ± 0.3	5.2 ± 0.3	5.4 ± 0.3	5.4 ± 0.3	5.9 ± 0.4
	Vertical R/nm	1.3 ± 0.3	2.8 ± 0.3	2.3 ± 0.3	2.5 ± 0.3	2.2 ± 0.3	2.0 ± 0.4	2.6 ± 0.3	3.1 ± 0.3	2.8 ± 0.3
	ρ/gcm^-3^	2.86	3.39	3.20	2.92	4.31	4.66	3.74	3.23	3.59
**e-beam**	Horizontal R/nm	3.3 ± 0.4	4.2 ± 0.6	8.3 ± 1.3	3.4 ± 0.4	3.5 ± 0.4	4.0 ± 0.3	5.2 ± 2.0	5.3 ± 3.7	6.1 ± 3.2
	Vertical R/nm	4.4 ± 0.7	4.1 ± 0.8	3.8 ± 0.9	4.4 ± 0.8	4.2 ± 0.8	4.7 ± 0.7	4.7 ± 1.6	6.6 ± 4.4	7.1 ± 4.1
	ρ/gcm^−3^	2.27	2.63	2.85	3.38	2.35	2.79	3.06	2.43	2.94

The CVD obtained films show lower density than the bulk density in anatase phase. The density value jumps prior to the phase transition and in rutile phase it decreases on approaching high temperatures. In the e-beam epitaxy obtained samples, the density is consistently lower than the bulk density in anatase phase as well as in the rutile phase. Our materials are porous, which is a desirable property for utilizing them in the second generation of the relatively cheap, efficient solar, Grätzel-type cells. The e-beam epitaxy obtained samples are more porous than the CVD obtained samples. At room temperature, the CVD and e-beam epitaxy obtained samples are 26% and 42% porous, respectively. The later has higher porosity, which is a better property to obtain the efficient dye-sensitized solar cell. In the case of galvanic cells of the second generation, the intercalation process is relevant and the obtained morphologies are under consideration for construction of these cells. The densities for CVD and e-beam epitaxy obtained samples are presented in Table 2.

These two morphologies of the TiO_2_ films are interesting for further investigation towards obtaining the most suitable average grain shapes and sizes for a possible application in opto-electronic devices.

## 4. Conclusions

By means of simultaneous SAXS/DSC/WAXD measurements, we have shown that the nanostructure of polymer electrolyte (PEO)_8_ZnCl_2_ can be modified by two treatments applied during preparation of the electrolytes: the irradiation by γ-rays and the addition of TiO_2_ nanosized particles. The significant role that the nanodimensions of the electrolyte material play in Zn^2+^-ion mobility, oxygen reduction in TiO_2_ nanograins, effect of confinement on polymer mobility and cross-linking are discussed. Both treatments largely enhanced the conductivity of the polymer electrolytes. SAXS information about the evolution of the average grain sizes during the phase transition gave insight into the nanomorphology, which influences the ionic transport in nanocomposite polymer electrolyte. Further optimization of the electrolyte properties are in progress as nanostructured materials are very attractive for batteries and other types of electronic devices.

As a further conclusion, the present study showed that 2D-GISAXS performed at the ELETTRA synchrotron light source can be applied for grain size determination in nanosized films of TiO_2_ on a glass substrate. Compared to other methods [10] of grain size determination, 2D-GISAXS is suitable for determination of grain sizes and even shape and distribution of nanograins. Nanosized TiO_2_ films are suitable materials for electrodes in new efficient solar cells and galvanic cells of second generation. We have a good path traced towards the proper morphology of the efficient electrodes in the new solar cells. A combination of the above discussed nanomaterials will be used in the construction of a new ecological galvanic and solar cells of the second generation.
